# Fluoroquinolone consumption and *Escherichia coli* resistance in Japan: an ecological study

**DOI:** 10.1186/s12889-019-6804-3

**Published:** 2019-04-23

**Authors:** Fumitaka Terahara, Hiroshi Nishiura

**Affiliations:** 1 Graduate School of Medicine, Hokkaido University, Kita 15 Jo Nishi 7 Chome, Kita-ku, Sapporo-shi, Hokkaido 060-8638 Japan; 2 CREST, Japan Science and Technology Agency, Honcho 4-1-8, Kawaguchi, Saitama, 332-0012 Japan

**Keywords:** Antibiotic resistance, *Escherichia coli*, Quinolones, Drug prescription, Epidemiology, Antimicrobial stewardship

## Abstract

**Background:**

The frequency of antimicrobial resistance has steadily increased worldwide, induced by inappropriate use of antibiotics in a variety of settings. We analyzed the ecological correlation between fluoroquinolone consumption and levofloxacin resistance in *Escherichia coli* in Japan.

**Methods:**

We collected information on cases of *E. coli* resistant to levofloxacin in 2015–2016 in all 47 prefectures from the Japan Nosocomial Infections Surveillance system. Information on fluoroquinolone consumption was obtained from pharmaceutical sales data. To address potential confounding, we also collected information on the number of physicians, nurses, and medical facilities per 100,000 individuals.

**Results:**

We identified higher fluoroquinolone consumption and higher resistance in western prefectures, and lower consumption and resistance in eastern prefectures. Multivariate analysis identified a positive correlation between fluoroquinolone consumption and levofloxacin resistance in both 2015 and 2016.

**Conclusions:**

Fluoroquinolone consumption and levofloxacin-resistant *E. coli* are potentially associated on a nationwide scale. The relationship between the two must be elucidated using additional studies with different epidemiological designs, so that any possible counter-measures, including alternative prescription, can be considered in the future.

## Background

The frequency of antimicrobial resistance (AMR) has steadily increased worldwide, induced by inappropriate use of antibiotics in a variety of settings [1]. Concurrent development of new antibiotics, however, has not necessarily been accelerated, rendering the issue of resistance a global concern. The World Health Organization endorsed the Global Action Plan on Antimicrobial Resistance in May 2015, encouraging all member countries to formulate national action plans within 2 years [1]. In response, Japan issued its National Action Plan on Antimicrobial Resistance in April 2016, explicitly specifying a number of priority goals to be achieved over the next 5 years to promote AMR measures [2]. Accordingly, various surveillance initiatives and resistance countermeasures are presently underway in the country.

In its Action Plan [2], Japan aims to lower the fraction of fluoroquinolone resistance in *Escherichia coli* from 45% in 2014 to less than 25% by 2020. Moreover, it aims to reduce daily fluoroquinolone use by 50% by 2020. *E. coli* belongs to the family *Enterobacteriaceae*, typically found in the lower gastrointestinal tract as microbial flora, and can cause urinary tract infection and sepsis if introduced into the bloodstream. β-lactam or quinolone antibiotics are frequently the first-choice treatment for *E. coli* infection [3]. Quinolones are highly bioavailable, permitting oral administration and good tissue distribution, and thus may be one of the most frequently used antibiotics at both outpatient and inpatient settings.

Increased use of antibiotics has been recognized as a major factor driving *E. coli* resistance over the last decades [4]. Fluoroquinolone resistance is easily established by point mutations in the DNA gyrase and via plasmid-mediated transfer [5], and various studies have indicated that the use of fluoroquinolone is most likely closely associated with the increase in fluoroquinolone resistance in *E. coli* [6–17]. Moreover, a few published studies reported that lowering the use of fluoroquinolone decreased the resistance proportion [18–20].

Published studies on the relationship between fluoroquinolone consumption and resistance in *E. coli*, however, have been mostly limited to specific healthcare facility settings, and findings on a national scale are yet to be reported. Moreover, the causal relationship must be carefully explored while adjusting for confounders. The purpose of the present study is to assess the ecological correlations between fluoroquinolone consumption and levofloxacin resistance in *E. coli* in Japan, using datasets from all 47 prefectures. Identifying the ecological association over the geographic space, one can further examine the causal link and underlying mechanisms that could potentially constitute future countermeasures.

## Methods

### Study design

The present study is an ecological study, exploring the relationship between fluoroquinolone consumption and resistance and exploiting geographic heterogeneities in the observed datasets in a cross-sectional manner, independently analyzing spatial datasets from 2015 and 2016. These two years were specifically examined, because only in these two years antimicrobial consumption data were accessible. For levofloxacin resistance, we collected the sensitivity testing results of levofloxacin whenever *E. coli* was detected from patients’ laboratory samples. The fluoroquinolone resistance was microbiologically judged by the current breakpoint of Clinical and Laboratory Standards Institute (CLSI) to define R (resistant) adhering to M100-S22. The information was retrieved from the clinical laboratory section of the Japan Nosocomial Infections Surveillance (JANIS) system [21] that encompasses the microbiological testing results of all registered medical facilities. Out of the total of medical facilities with 500 beds or greater, 77.1 and 80.9% of eligible hospitals contribute to the surveillance in 2015 and 2016, respectively. Among all hospitals with 200 beds or greater, 44.5 and 48.0% of the total are registered to JANIS in 2015 and 2016. Smaller hospitals with 200 or fewer beds do not usually have an independent microbiological laboratory, and thus, only 4.4 and 6.6% of the total are registered in 2015 and 2016, respectively. All datasets originated from microbiological testing at inpatient service. The corresponding data are the sum of all relevant testing results from both outpatient and inpatient services regardless of characteristics such as infection status, colonization, or carrier.

For fluoroquinolone consumption, we used pharmaceutical sales data from wholesalers, representing the total amount of drug sold [22] by prefecture in 2015–2016, which has been publicly shared by the AMR Clinical Reference Centre, the National Centre for Global Health and Medicine. The sales data were standardized in accordance with the Anatomical Therapeutic Chemical classification using defined daily dose as a measurement unit, as recommended by the World Health Organization Collaborating Centre for Drug Statistics and Methodology [23]. The population-weighted consumption of oral and parenteral antimicrobials was expressed as defined daily dose per 1000 inhabitants per day (DID).

As potential confounders, we also collected the following information by prefecture for 2015–2016, and used them as explanatory variables: (i) The number of physicians per 100,000 individuals [24], (ii) the number of nurses per 100,000 individuals [25], (iii) the numbers of hospitals and clinics per 100,000 individuals [26], (iv) the number of nursing homes per 100,000 individuals [27], (v) the number of registered medical facilities in JANIS and the fraction of registered medical facilities with 500 or more inpatient beds [21], (vi) the fraction of elderly individuals in the population [28], and (vii) the average length of a hospital stay [29].

### Statistical analysis

We first assessed the descriptive features of the examined variables, their distributions and summary statistics. As some variables yielded a variance-to-mean ratio greatly exceeding 1, we consistently examined median and interquartile ranges (IQR) to summarize the distribution of all variables. For some variables with skewed distributions, we log-transformed the data to allow for an approximate normal distribution. Next, we examined univariate relationships as ecological correlations between two continuous variables, namely the fraction of levofloxacin-resistant *E. coli* cases reported and another variable. The univariate linear regression method was used to test the correlation. Finally, to adjust for confounding variables, we used multiple linear regression. Two methods were used for selecting variables to be included in the model: We included all significantly correlated variables in the multivariate model in the first method, and in the second method, a backward stepwise approach was used, applying the minimum Bayesian information criterion to select variables in the final model. The statistically significant level was set at α = 0.05. All statistical data were analyzed with JMP Pro version 14.0 (SAS Institute Inc., Cary, NC, USA).

## Results

**Fig. 1:**
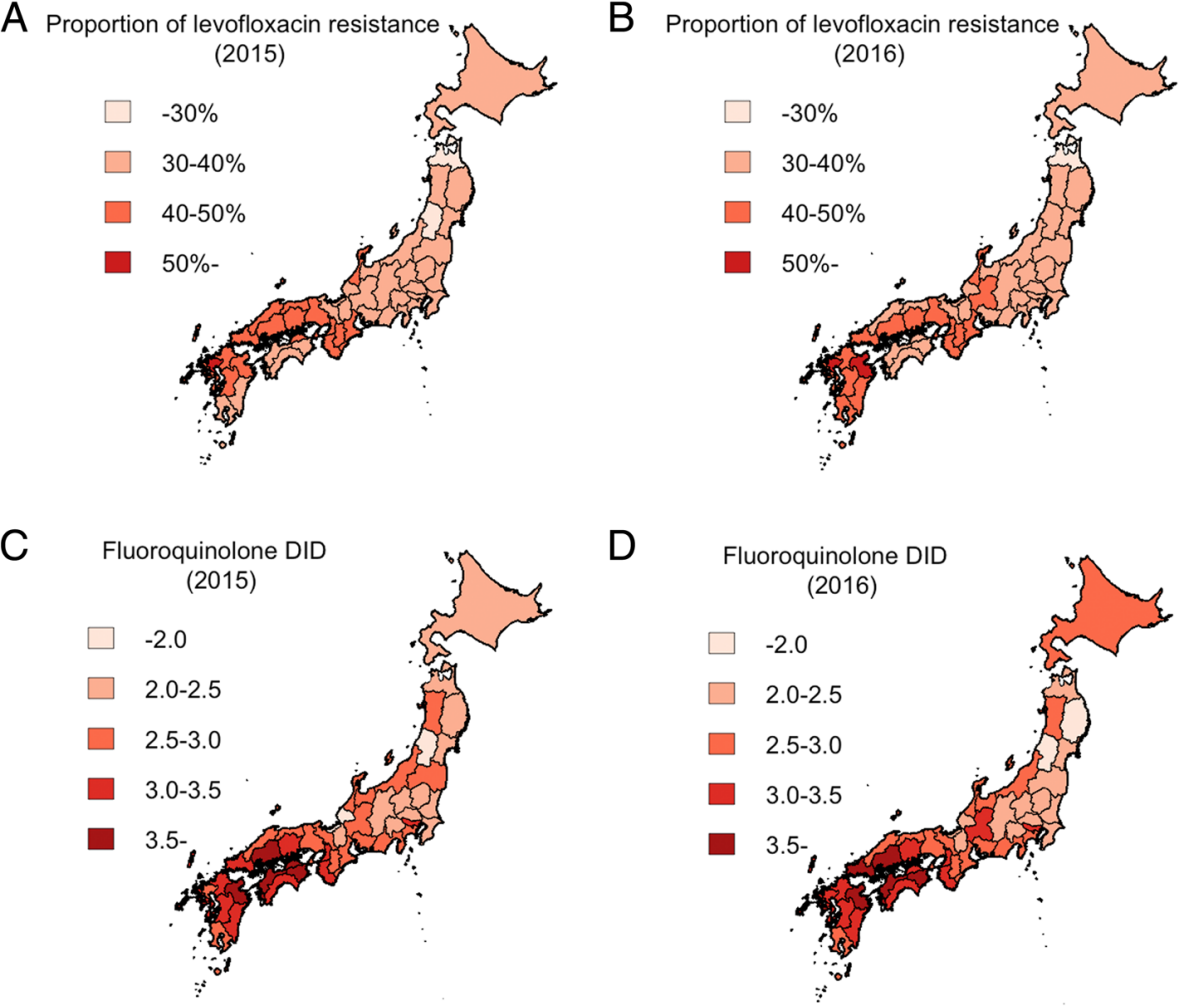
Geographic distribution of fluoroquinolone consumption and levofloxacin resistance in *Escherichia coli* in Japan by prefecture, 2015–2016. Proportion of *E. coli* resistant to levofloxacin in 2015 (**a**) and 2016 (**b**). Fluoroquinolone consumption in 2015 (**c**) and 2016 (**d**), as defined daily dose per 1000 inhabitants per day (DID)

Table 1 shows the summary statistics of the variables examined that were considered potentially associated with the geographic heterogeneity of levofloxacin-resistant *E. coli* proportions. In 2015 and 2016, median values (and IQR) of the total isolates of *E. coli* by prefecture were 4017 (IQR: 2141-6333) and 3969 (IQR: 2744-7133), respectively. Out of those samples of *E. coli* isolates, the overall proportion of levofloxacin-resistant *E. coli* (hereafter, referred to as the “resistance proportion”) was 37.4% in 2015 and 38.3% in 2016. Median consumption of fluoroquinolones was 2.8 DID in both 2015 and 2016. Of the total prescriptions, the proportion of orally administered fluoroquinolones was 98.6% in 2015 and 98.5% in 2016, respectively. Figure 1 shows the geographic distributions of fluoroquinolone consumption and levofloxacin resistance. Among the 47 prefectures in Japan, the proportion of levofloxacin resistance in *E. coli* was overall lower in the eastern region than in the west, with the lowest proportions estimated in Yamagata and Aomori (27.1 and 26.2%, in 2015 and 2016, respectively; Fig. 1a and b). The highest proportion was seen in the western Saga prefecture, and estimated at 51.1 and 55.0% in 2015 and 2016, respectively. Similarly, fluoroquinolone consumption was lower in eastern prefectures than in the west on a whole, and the lowest values were observed for Yamagata and Fukui. The highest value of fluoroquinolone consumption was observed in Tokushima, a western prefecture (Fig. 1c and d).

**Table 1:** Descriptive characteristics of variables used as statistical determinants of levofloxacin resistance in *Escherichia coli* in Japan, 2015–2016

Variable	Median (IQR)	
	2015	2016
Proportion of resistance (%)	37.4 (33.9–42.0)	38.3 (35.5–42.6)
Fluoroquinolone consumption (DID)	2.8 (2.4–3.2)	2.8 (2.4–3.3)
Number of physicians per 100,000 individuals	242.4 (217.0–279.8)	242.4 (217.0–279.8)
Number of nurses per 100,000 individuals	965.5 (817.0–1104.2)	965.5 (817.0–1104.2)
Number of hospitals per 100,000 individuals	7.1 (5.7–10.0)	7.2 (5.7–10.0)
Number of clinics per 100,000 individuals	81.1 (72.8–90.2)	81.3 (90.7–72.7)
Number of nursing homes per 100,000 individuals	7.3 (6.2–8.3)	6.9 (5.8–8.1)
Number of medical facilities included in surveillance	21.0 (15.0–37.0)	26.0 (17.0–40.0)
Proportion of medical facilities with 500 or more beds (%)	20.0 (15.8–27.7)	17.9 (12.5–25.0)
Proportion of elderly in the population (%)	28.7 (26.8–30.1)	28.7 (26.8–30.1)
Average length of hospital stay (days)	32.0 (29.2–34.1)	31.8 (28.8–33.8)

*DID* Defined daily dose per 1000 inhabitants per day, *IQR* Interquartile range

**Fig. 2:**
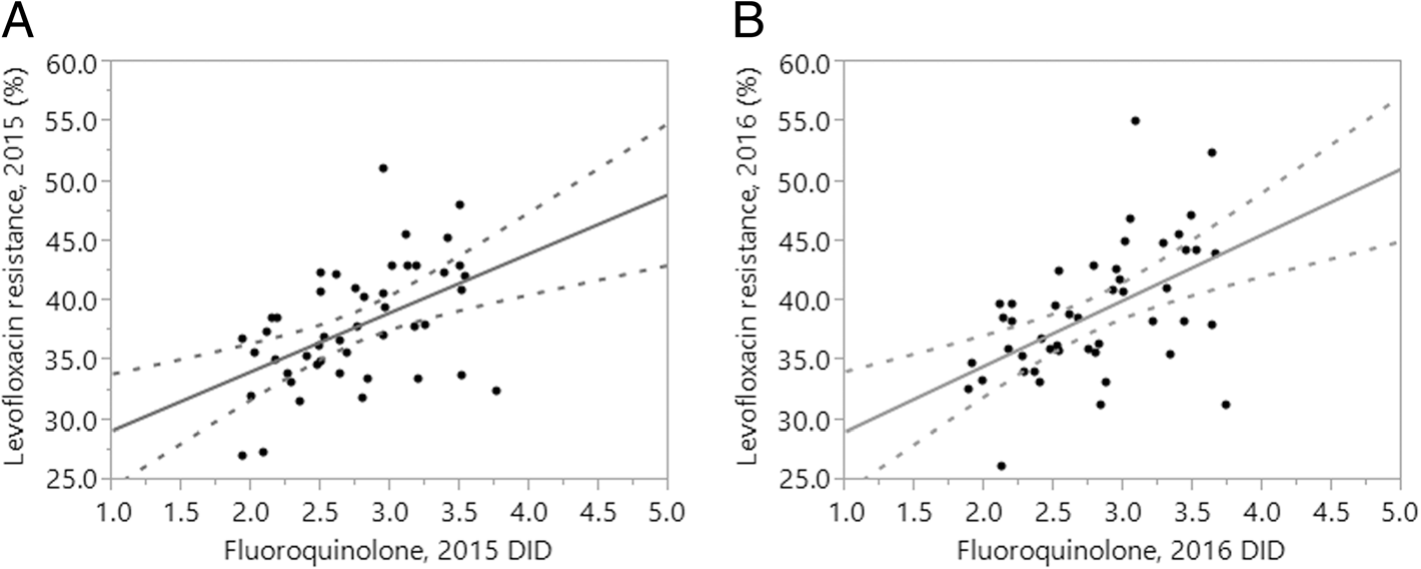
Univariate correlation between fluoroquinolone consumption and levofloxacin resistance in *Escherichia coli* in Japan, 2015–2016. Correlation between fluoroquinolone consumption and the proportion of *E. coli* resistant to levofloxacin in 2015 (**a**) and 2016 (**b**), as defined daily dose per 1000 inhabitants per day (DID)

Table 2 summarizes the results of univariate correlation by year. The following variables were consistently and positively correlated with the proportion of levofloxacin resistance: Fluoroquinolone DID, the number of physicians, the number of nurses, and the number of hospitals and clinics. In contrast, the proportion of levofloxacin resistance was not significantly correlated with the number of nursing homes, the number of medical facilities registered in JANIS, the proportion of registered facilities with 500 or more beds, the average length of hospital stay, or the proportion of elderly individuals in the population. Figure 2 shows the crude univariate correlation between consumption of fluoroquinolone and the proportion of levofloxacin resistance. The linear regression coefficient was estimated at 0.50 and 0.52 for 2015 and 2016, respectively.

**Table 2:** Univariate correlations between the proportion of levofloxacin-resistant *Esherichia coli* and selected variables in Japan, 2015–2016

Variable	Proportion resistant in 2015		Proportion resistant in 2016	
	Coefficient	*p* value	Coefficient	*p* value
Fluoroquinolone consumption (DID)	0.50	< 0.01	0.52	< 0.01
Number of physicians per 100,000 individuals	0.49	< 0.01	0.42	< 0.01
Number of nurses per 100,000 individuals	0.36	0.01	0.31	0.03
Number of hospitals per 100,000 individuals^a^	0.34	0.02	0.32	0.03
Number of clinics per 100,000 individuals	0.43	< 0.01	0.41	< 0.01
Number of nursing homes per 100,000 individuals	−0.04	0.80	−0.17	0.26
Number of medical facilities included in surveillance^a^	−0.03	0.84	0.05	0.72
Proportion of medical facilities with 500 or more beds (%)^a^	−0.10	0.52	−0.02	0.88
Average length of hospital stay (days)	0.27	0.07	0.29	0.05
Proportion of elderly in the population (%)	0.04	0.79	−0.04	0.79

*DID* Defined daily dose per 1000 inhabitants per day. ^a^Indicates log-transformed data

Table 3 shows the results from multivariate analyses. In advance of conducting multivariate linear regression, we identified a strong positive cross-correlation between fluoroquinolone consumption and the number of physicians (regression coefficient = 0.008 for both 2015 and 2016 for each one-physician increase per 100,000 individuals; *p* < 0.001). We therefore used the product of these two variables as an explanatory variable to address the possible interaction. When including all variables that were significantly correlated in the univariate analysis in the final multivariate model, no significant variables remained in the final model for either 2015 or 2016 data, and fluoroquinolone consumption was not significant (*p* = 0.09) with a large effect at 19.3 per DID increase in 2015, and not statistically significant (*p* = 0.16) with a large effect at 17.1 per DID increase in 2016. Employing the stepwise method, only fluoroquinolone consumption remained in the final model, with 5.0 and 5.5 per DID increase in 2015 and 2016, respectively (*p* < 0.01 for both years). The coefficient of determination was estimated at 0.25 for 2015 and 0.27 for 2016, with F-values of 14.8 and 17.0, respectively (*p* < 0.01 for both years), reflecting an overall success in identifying at least some factors involved in levofloxacin resistance.

**Table 3:** Multiple regression analysis of the proportion of levofloxacin-resistant *Escherichia coli* in Japan, 2015–2016

Variable	Proportion resistant in 2015				Proportion resistant in 2016			
	Partial regression coefficient	SE	t	*p* value	Partial regression coefficient	SE	t	*p* value
Intercept constant	−23.8	29.8	−0.80	0.43	−14.8	32.9	−0.45	0.66
Fluoroquinolone consumption (DID)	19.3	11.1	1.75	0.09	17.1	12.0	1.43	0.16
Number of physicians per 100,000 individuals	0.2	0.1	1.71	0.10	0.2	0.1	1.14	0.26
Number of nurses per 100,000 individuals	−0.1<	< 0.1	−0.39	0.70	− 0.1<	< 0.1	− 0.37	0.71
Number of hospitals per 100,000 individuals	1.5	4.2	0.36	0.72	2.0	4.8	0.41	0.69
Number of clinics per 100,000 individuals	< 0.1	0.1	0.06	0.96	< 0.1	0.1	0.35	0.73
Dummy variable (fluoroquinolone consumption [DID] × the number of physicians)	−0.1	< 0.1	−1.49	0.14	−0.1	< 0.1	−1.08	0.29

2015: *R*^2^ = 0.34, *F* = 3.40, *p* < 0.01

2016: *R*^2^ = 0.31, *F* = 3.00, *p* = 0.02

*DID* Defined daily dose per 1000 inhabitants per day, *SE* Standard error

## Discussion

The present study examined the ecological correlation between fluoroquinolone consumption and levofloxacin-resistant *E. coli* in Japan, adjusting for potential confounding variables. Comparing fluoroquinolone consumption and levofloxacin resistance by prefecture across Japan, we identified relatively higher values in the western region and lower values in the eastern region. To explain the underlying mechanisms of such geographic heterogeneities, we conducted univariate analyses, and identified not only fluoroquinolone consumption but also the numbers of physicians, nurses, hospitals, and clinics per 100,000 individuals as positively correlated with the proportion of levofloxacin-resistant *E. coli*. Multivariate analyses validated the positive correlation between fluoroquinolone consumption and levofloxacin resistance.

The present study’s largest contribution is its demonstration of the link between fluoroquinolone consumption and levofloxacin resistance using nationwide surveillance data, while appropriately addressing potential confounding variables. Our study rested on a digitalized laboratory-based surveillance system, which covers 20% of healthcare facilities and 80% of medical facilities with 500 or more beds; to our knowledge, no published study has explored this relationship using a comparably large national-scale dataset. Antibiotic consumption has already been shown to be positively and strongly correlated with prescription [30], and thus, our study endorses published conclusions [6–17] that the prescription of fluoroquinolones induces the emergence (and perhaps maintenance) of resistant *E. coli*. Various factors may contribute to the development of levofloxacin resistance in *E. coli*, but our study did not identify any significant healthcare services-associated variables that were correlated with levofloxacin resistance. A high density of physicians could lead to competition and reduce hospital visits by individuals feeling unwell, in turn promoting inappropriate (and increased) prescription of fluoroquinolones. This notion was in line with the univariate analysis, but the number of physicians was removed in the final multivariate model, indicating that the information that was contained in our dataset (i.e. only the variations among the 47 prefectures) was not sufficient to establish a relationship between the number of physicians per 100,000 individuals and levofloxacin resistance. Only fluoroquinolone consumption was left in the final multivariate model using the stepwise method, as this variable is a strong predictor of geographic heterogeneity of levofloxacin-resistant *E. coli*.

An important implication of the correlation between fluoroquinolone consumption and levofloxacin resistance in *E. coli* is that the restricted use of fluoroquinolones could potentially contribute to lowering the resistance proportion, if the resistant *E. coli* is evolutionarily unfit. The recovery of sensitivity at the population level depends on the fitness cost (i.e. transmission and self-maintenance capacity) of resistant *E. coli*. The cross-sectional design has ignored two important aspects of the emergence: (i) The induction period, i.e. the time from prescription to an increase in resistant *E. coli*, and (ii) the length of survival of resistant *E. coli* as part of the human gut microflora or elsewhere [31]. In addition to these known factors, it is worth noting that cross-resistance from the use of other antibiotics is also indicated [11, 32, 33]. The population benefit of restricted prescription requires additional epidemiological evidence and analyses. In particular, the present study was not able to account for disease burden that is associated with resistant *E. coli*, including urinary tract infections and respiratory tract infections, because they are not categorized as notifiable diseases in Japan. The recovery of sensitivity to fluoroquinolones should ideally be attributed to the reduction in those disease burden estimates via an appropriate epidemiological study design.

In Japan, medical expenditure has been shown to exhibit geographically heterogeneous patterns, mostly indicating higher expenditure in the west and lower in the east [34]. We have shown that fluoroquinolone consumption and resistance are no exceptions: Despite the management of healthcare services under the same system, such geographic heterogeneities are evidently observed, and no clear mechanisms were identified to explain the differences. Our study demonstrated that geographic heterogeneity of levofloxacin resistance was most likely regulated by that of fluoroquinolone consumption, and the mechanisms underlying the increase in resistance should be determined in the context of antimicrobial stewardship [35–40]. A top-down approach to restricting inappropriate prescription of antibiotics may yield evidence of the causal link suggested by our analyses.

Several limitations must be noted. First, the present study rests on an ecological study design that is known to be prone to unobserved confounding. What has been identified does not immediately lead to a causal link at the individual level. Second, the JANIS data that we mined were not accompanied by information on the type of clinical specimen or sensitivity to other antimicrobial agents. More detailed insights into levofloxacin resistance could be gained by exploring a body part from which the samples were obtained, and also examining drug sensitivity of the same bacterium to other antimicrobial agents. Third, antimicrobial consumption was measured in the present study by defined daily dose, but this approach overlooks individual heterogeneities in the prescribed dose (e.g. a lower dose in children). Published studies at the individual level can overcome this problem. Fourth, we used the number of physicians as one of variables, but the number was not stratified by hospital and primary care physicians. For the entire Japan, we have an access to the total count, i.e., there are 101,884 physicians working for clinics out of the total of 296,845 (34.3%), but we did not have an access to the corresponding numbers by prefecture. Fifth, we used consumption and resistant frequency data, but the fluoroquinolone use at outpatient and inpatient pharmaceutical sales were not separately recorded, and moreover, microbial samples were also not stratified by inpatient and outpatient services. The distinction has an important implication for considering future restriction of antimicrobial use. Sixth, we examined only 2015 and 2016 data, because of limited access to antimicrobial consumption data. The longer the data series are collected, the more plausible that we will have a chance to elucidate the causal link, especially in the case fluoroquinolone use may be restricted from some future point in time.

In conclusion, we showed an ecological correlation between fluoroquinolone consumption and levofloxacin resistance in Japan, analyzing prefectural datasets from two recent years. The geospatial pattern of fluoroquinolone use correlated with that of resistance. The relationship between the two must be elucidated using additional studies with different epidemiological designs, so that any possible countermeasures, including alternative prescription, can be considered in the future.

## Conclusions

Fluoroquinolone consumption and levofloxacin-resistant *E. coli* are potentially associated on a nationwide scale. The relationship between the two must be elucidated using additional studies with different epidemiological designs, so that any possible counter-measures, including alternative prescription, can be considered in the future.

## Abbreviations

AMR: antimicrobial resistance
DID: defined daily dose per 1000 inhabitants per day
*E. coli*: *Escherichia coli*
IQR: Interquartile range
JANIS: Japan Nosocomial Infections Surveillance
